# Antioxidant Activity of Maillard Reaction Products in Dairy Products: Formation, Influencing Factors, and Applications

**DOI:** 10.3390/foods15020351

**Published:** 2026-01-18

**Authors:** Hong Lan, Jinjing Xu, Xiaolong Lu, Xinyue Hu, Liteng Peng, Qingyou Liu, Fei Ye, Hao Qi

**Affiliations:** 1 Guangdong Provincial Key Laboratory of Animal Molecular Design and Precise Breeding, School of Animal Science and Technology, Foshan University, Foshan 528200, China; honglan1909@163.com (H.L.); 17638085662@163.com (J.X.); luxiaolong2001@163.com (X.L.); huxinyue21@163.com (X.H.); 13138266763@163.com (L.P.); qyliu-gene@fosu.edu.cn (Q.L.); 2 Analysis and Testing Center of Foshan University, Foshan University, Foshan 528200, China; yefei08312@fosu.edu.cn

**Keywords:** natural antioxidants, milk, melanoidins, reductones, volatile heterocyclic compounds

## Abstract

Dairy products contain complex types and contents of proteins, lipids, and lactose. The Maillard reaction (MR) occurs between proteins and reducing sugars during the processing and storage of dairy products. Maillard reaction products (MRPs) have garnered attention for their potential antioxidant activity. MRPs include melanoidins, reductones, and volatile heterocyclic compounds, which affect flavor and color. Relevant literature was identified through a structured search of PubMed and Web of Science; studies were included if they investigated MRPs in dairy products and reported antioxidant-related outcomes. This review offers a comprehensive overview of the MR in dairy products, systematically investigating the influence of protein, reducing sugars, and their ratios, as well as reaction conditions (process technology, temperature, time, pH, and water activity) on the formation and antioxidant activity of MRPs. The review also covers current applications and the future potential of MRPs as natural antioxidants in dairy products. Although MRPs effectively delay lipid oxidation and enhance stability in dairy products, research on their molecular structure and antioxidant mechanisms remains insufficient. Future research should focus on understanding the multifactorial synergistic effects within the complex dairy matrix, elucidating the molecular structure and extraction of antioxidant substances, and developing regulatory techniques to balance the antioxidant properties of MRPs with the safety concerns of potential harmful byproducts.

## 1. Introduction

Dairy products provide essential nutrients like lipids, lactose, proteins, and vitamins. They are susceptible to oxidative deterioration during dairy processing and storage, leading to rancidity, nutrient loss, and reduced shelf life. To reduce these effects, synthetic antioxidants like butylated hydroxyanisole (BHA) and butylated hydroxytoluene (BHT) are commonly used in food systems. However, rising consumer concerns about the potential health risks linked to synthetic additives have fostered a growing interest in the development of safe, effective, and naturally derived antioxidant alternatives [1,2]. Natural antioxidants are primarily obtained from natural edible sources. Common natural antioxidants found in dairy products include vitamin C, vitamin E, carotenoids, and phenolic compounds, which are known for their high safety and low toxicity [3,4]. These inherent natural antioxidant components within dairy products are prone to degradation during processing and storage [5]. Under the same conditions, the Maillard reaction (MR) generally proceeds, leading to the formation of Maillard reaction products (MRPs) that can partially or even fully compensate for the loss of antioxidant capacity, thereby enhancing the overall antioxidative potential of the system [5,6].

MR, also known as the carbonyl–amine reaction, was first discovered by French biochemist Louis-Camille Maillard in 1912 [7]. In 1953, Hodge proposed the first coherent mechanism of the MR [8]. This reaction primarily occurs between carbonyl compounds (e.g., reducing sugars) and amino compounds (e.g., amino acids and proteins) [7]. MRPs not only influence the flavor and color of dairy products but also exhibit multiple biological activities, including antioxidant, antimicrobial activity, and anti-inflammatory effects, as well as memory enhancement [1,7,9,10,11]. In 1954, Iwainsky and Franzke first reported the antioxidative effect of MRPs, laying the foundation for subsequent research [12].

Raw milk contains almost no MRPs [13]. But during the processing and storage, treatments such as thermal processing, ultrasonication, microwave heating, and high hydrostatic pressure may induce MR [14,15,16]. MRPs including melanoidin-like compounds, reductones, and a range of nitrogen- and sulfur-containing volatile heterocyclic compounds, exhibiting varying levels of antioxidant activity. Certain MRPs exhibit antioxidant activity comparable to those of synthetic antioxidants [1,17]. These compounds effectively scavenge free radicals and prevent lipid oxidation, thereby delaying oxidative rancidity, color change, and nutrient loss [10]. Consequently, as naturally formed antioxidants during processing and storage, MRPs provide promising candidates for developing safe and effective antioxidant strategies. This review focuses on MRPs within the dairy system, systematically examining their antioxidant activity through a three-part framework: formation mechanisms, influencing factors, and applications. Unlike previous studies that primarily addressed model systems or general food systems (Table 1), this review focuses on the antioxidant activity of MRPs derived from dairy products and the effects of reaction conditions. This provides a theoretical basis for quality control of antioxidants during dairy processing and the development of natural antioxidants.

## 2. Methods

### 2.1. Search Strategy

Following the Preferred Reporting Items for Systematic Reviews and Meta-Analyses (PRISMA 2020) guidelines, a systematic literature search was conducted in PubMed and Web of Science [21,22,23]. Using search combined keywords (see Table 2) and Boolean operators (AND, OR), we aimed to identify original research papers and review articles in English on milk, dairy products, the Maillard reaction, and antioxidant activity, with no date restrictions. Complete search strategies and results for each database, including all search terms and applied restrictions, are provided in the Supplementary Materials. All literature records were imported into Excel for screening management. Two reviewers independently screened titles, abstracts, and full texts against inclusion criteria. Discrepancies were resolved through discussion or by a third reviewer. Included articles covered core topics: Maillard reaction mechanisms, Maillard reactions in protein-carbohydrate models, and methods for assessing antioxidant activity.

### 2.2. Eligibility Criteria

#### 2.2.1. Inclusion Criteria

Studies were included if they met the following criteria: (a) No time restrictions on selected literature; (b) Published in English to ensure accuracy and reliability of research methods, results data, and interpretation of conclusions; (c) Original research articles; (d) Employed comparative experimental designs (e.g., groups with different reaction conditions or different substrates) or analytical designs explicitly focused on the Maillard reaction in dairy systems, and included measurements of antioxidant activity-related indicators; (e) The study subjects were dairy products (e.g., milk, yogurt, milk powder, cheese) or milk protein/amino acid-reducing sugar model systems, and the research findings were directly related to the Maillard reaction during dairy processing and storage.

#### 2.2.2. Exclusion Criteria

**Figure 1:**
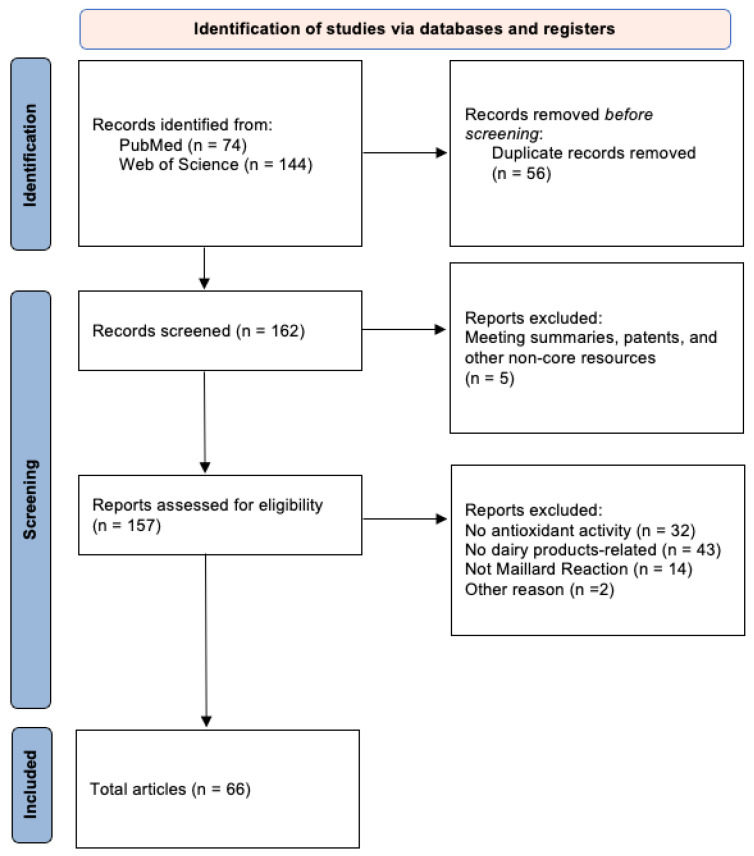
PRISMA (Preferred Reporting Items for Systematic Reviews and Meta-Analyses) flow diagram for studies retrieved through the searching and selection process.

The following studies will be excluded: (a) Studies on Maillard reactions involving non-dairy matrices or non-dairy proteins/reducing sugars (e.g., studies related to plant proteins); (b) Studies that did not measure antioxidant activity indicators; (c) Non-core research resources such as conference abstracts or patents, unless irreplaceable core data exists and is highly relevant—no such instances were found in this study; (d) Duplicate records across databases (if the same study appears in multiple databases, only one complete record is retained). The PRISMA guidelines form the basis for paper screening, as illustrated in the flowchart (Figure 1).

## 3. The Maillard Reaction Process in Dairy Products

### 3.1. Early Stage: Glycation and Rearrangement

**Figure 2:**
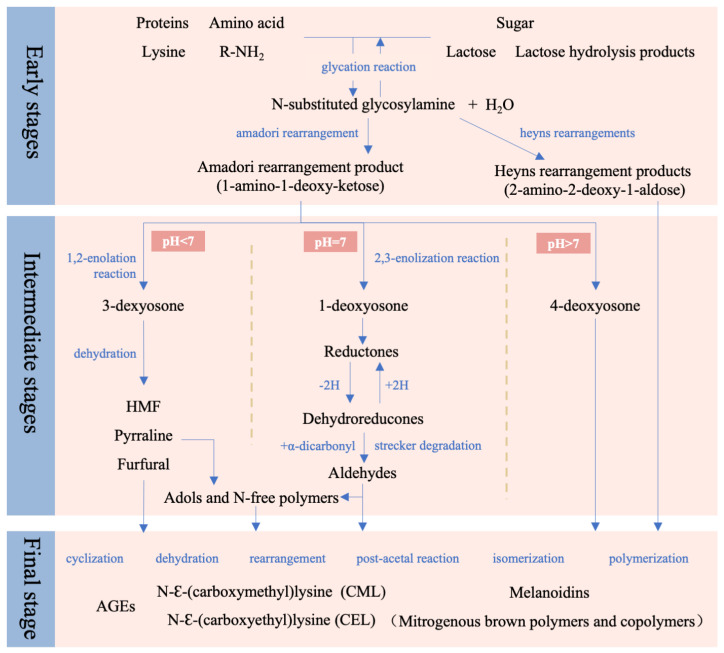
Maillard reaction process in dairy products.

In the early stage, a condensation reaction between the amino groups of proteins or amino acids and the carbonyl groups of reducing sugars results in the formation of an unstable Schiff base (N-substituted glycosylamine) with the removal of a water molecule [24]. This glycation step is the only reversible part of MR [1,25]. As shown in Figure 2, the type of reducing sugar determines the rearrangement pathway and the intermediates produced [26]. The N-glycosylamine generated from aldose undergoes Amadori rearrangement, yielding 1-amino-1-deoxy-2-ketose. Lysine participates in the Amadori rearrangement to produce N^ε^-(1-deoxy-D-lactulos-1-yl)-Lys (also known as lactulosyl-lysine) [24]. Ketose undergoes a Heyns rearrangement to produce 2-amino-2-deoxy-1-aldose. In addition, dairy products containing hydrolyzed lactose include highly reactive monosaccharides (glucose and galactose), which can react with amino acid residues to generate lactulosyl-lysine and tagulosyl-lysine [27].

### 3.2. Intermediate Stage: Degradation and Fragmentation

In the intermediate stage, Amadori rearrangement products (ARPs) undergo further degradation through a series of reactions such as sugar dehydration, deamination, Strecker degradation, and sugar fragmentation [1]. Based on the pH value, three main reaction pathways are discovered (Figure 2). Under acidic conditions, 1,2-enolization predominates, producing 3-deoxyosones, which subsequently undergo dehydration to yield 5-hydroxymethylfurfural (HMF), pyrraline, and furfural. Furthermore, under such conditions, lactulosyl-lysine can be hydrolyzed to form ε-N-(2-furoylmethyl)-L-lysine (furosine) [28]. Under alkaline conditions, Amadori compounds undergo cleavage to generate 4-deoxyosones [1,7,29,30]. Under neutral conditions, 2,3-enolization predominates, leading to the formation of 1-deoxyosones, which are subsequently converted into reductones and dehydroreductones. The latter undergo Strecker degradation to produce aldehydes. Considering that milk and common dairy systems typically have near-neutral pH and contain lactose, the 2,3-enolization pathway is regarded as the dominant reaction route.

### 3.3. Final Stage: Polymerization and Melanoidin Formation

In the final stage, low-molecular-weight intermediates undergo a series of complex reactions including cyclization, dehydration, aldol-type condensation, and isomerization, resulting in the formation of advanced glycation end-products (AGEs), such as N-ε-(carboxymethyl)lysine (CML) and N-ε-(carboxyethyl)lysine (CEL), which are representative compounds of this stage [1,7,20,30,31]. Concurrently, brown, nitrogen-rich macromolecules known as melanoidins are synthesized through complex polycondensation processes [24].

## 4. Research Methods Related to the Maillard Reaction

### 4.1. Identification of Maillard Reaction Stages

The progression of MR can be characterized by the color changes and ultraviolet (UV) absorption properties of MRPs. The early-stage products of the MR are colorless and exhibit no UV absorption. In the intermediate stage, MRPs appear colorless to pale yellow, with formation typically monitored by absorbance at 294 nm. In contrast, brown MRPs generated in the final stage are quantified based on absorbance at 420 nm [13,28,32]. UV–vis spectra detection is susceptible to interference from other chromophores [33]. In addition, changes in the concentration of HMF are frequently used to evaluate the extent of the MR during milk heat treatment and storage [34]. The content of free amino acids decreases after participating in MR. Therefore, the loss of free amino groups determined using the o-phthaldialdehyde (OPA) method can also serve as an indicator of Maillard reaction intensity [35,36].

### 4.2. Determination of Maillard Reaction Products

Melanoidins poses challenges for direct quantification and structural characterization due to its high molecular weight, structural diversity, and non-volatility. Traditional detection methods for melanoidins primarily rely on characterizing its overall physicochemical properties, such as the browning index, ultraviolet-visible absorption spectra, and fluorescence detection [37]. However, the above traditional methods lack specificity. Due to their diverse formation pathways and complex structures, no reliable method currently exists for accurate structure of melanoidins [38]. However, by selectively separating small molecules, macromolecules, and melanin-like substances using macroporous resin adsorption technology, and employing techniques such as Fourier Transform Infrared Spectroscopy (FT-IR) and Nuclear Magnetic Resonance (NMR) to detect functional groups and heterocyclic frameworks within melanoidins [39]. In recent years, metabolomics has gained popularity in food research. Emilia Pogoda utilized Liquid Chromatography-Mass Spectrometry (LC-MS) technology to identify metabolites associated with the antioxidant activity of buckwheat honey [40].Although research on melanoidins in dairy matrices remains limited, these methodologies provide a reference for detection in milk systems.

Reductones is an intermediate formed during the Maillard reaction, characterized by high instability and transience [41]. Reductones and their related intermediates fall under the category of α-dicarbonyl compounds and can be detected and quantified using LC-MS/MS coupled with either derivatization or non-derivatization methods. Although modern literature specifically targeting reductones is limited, detection strategies for α-dicarbonyls are relatively common in the analysis of Maillard intermediates [16,42]. Common derivatization agents include o-phenylenediamine (OPD) and o-(2,3,4,5,6-pentafluorobenzyl)hydroxylamine (PFBHA), followed by liquid chromatography or gas chromatography analysis, supplemented with mass spectrometry or fluorescence detection [43].

Volatile MPRs include nitrogen-, oxygen-, and sulfur-containing heterocyclic compounds such as pyrazines, furans, thiophenes, and pyrroles [44]. Modern analysis of these volatile MRPs typically employs headspace sampling techniques—such as headspace solid-phase microextraction (HS-SPME) coupled with gas chromatography-mass spectrometry (GC-MS)—to capture and quantify trace volatile compounds. This method is simple to operate and requires no pretreatment such as solvent extraction. A study has applied E-nose and GC–MS/IMS to reveal the yogurt-like and baked aroma notes of Maillard brown soy yogurt (MBSY), demonstrating the potential of the Maillard reaction in enhancing the functionality and flavor profiling of plant-based dairy products [45].

### 4.3. Antioxidant Activity Assessment Methods of Maillard Reaction Products

#### 4.3.1. Total Reducing Power Assays

Ferric reducing antioxidant power (FRAP) assay measures the total reducing capacity of antioxidants by converting the Fe^3+^-TPTZ complex (pale yellow) to the Fe^2+^-TPTZ complex (blue) (Table 3) [46]. Its absorbance at 593 nm is proportional to the reducing power, thereby enabling quantitative comparison of antioxidant capacity among samples. The FRAP method is relatively simple, quick, sensitive, and inexpensive to perform [47]. However, FRAP measurements of antioxidant components are typically conducted under low pH conditions, rendering them susceptible to interference from milk protein precipitation. To address this, Jayendra K. Amamcharla proposed an improved FRAP method employing a syringe to remove precipitated milk proteins, reducing the %RSD to between 1.5 and 4.4% [46].

#### 4.3.2. Radical Scavenging Assays

MRPs have been demonstrated to possess antioxidant properties and free radical scavenging capabilities [7]. 2,2-Diphenyl-1-picrylhydrazyl (DPPH) and 2,2′-azino-bis(3-ethylbenzthiazoline-6-sulfonic acid) (ABTS) are widely used to evaluate the total antioxidant activity of milk and dairy products [3,14,48]. As shown in Table 3, the DPPH and ABTS methods depend on the color change in redox indicators to determine antioxidant capacity [49,50]. The DPPH method offers the advantages of being fast and simple, but it has certain limitations when samples with weak or no lipophilicity [51]. The ABTS method is suitable for the determination of both water-soluble and fat-soluble antioxidants [52,53]. Compared to the conventional peroxide value (POV) method for assessing primary oxidation in milk lipids, the DPPH and FRAP assays are more sensitive and thus better suited for evaluating the antioxidant capacity of dairy products [14]. No single method can comprehensively evaluate the antioxidant capacity of complex dairy products; it is typically necessary to combine multiple methods based on different principles (such as FRAP, DPPH, etc.) for a comprehensive assessment.

### 4.4. Model System

Analytical methods measure the total antioxidant capacity of the sample and cannot distinguish the contribution of individual compounds. Due to the complex composition of dairy products, which contain various naturally occurring antioxidant substances, it is challenging to quantify the specific contribution of MRPs [54]. To address this issue, model systems have been developed to simplify the research by focusing on the basic components of dairy products. Such models help eliminate interference from other active substances and allow for targeted investigation of key MRPs [26]. Indeed, most available data on the MR have been derived from sugar–amino acid or sugar–protein model systems [55,56].

Cortés Yáñez et al. found that the positive correlation trend between the “reaction stage and antioxidant activity” in the model system corresponds to that observed in skim milk powder, sweetened condensed milk, and Dulce de Leche [5]. Models and real dairy products may exhibit similar trends, but antioxidant indicators derived from models will exhibit certain shifts when applied to real dairy products. The direction of these shifts depends on determining factors such as dairy product type, and reaction conditions. Model systems typically incorporate only two or a few reactants, whereas real dairy products contain a richer array of reactive components and their associated reaction sites. The concentration of reaction sites varies across different dairy components [57,58]. The simplified composition of model systems struggles to capture this multi-component, multi-site complexity, leading to discrepancies in MR intensity, product formation, and antioxidant performance. Furthermore, the interactions between structure and composition in real milk matrices are more intricate, significantly altering the accessibility of reactive sites. In dairy products, the encounter between lactose and protein reactive sites is constrained by protein spatial conformation, colloidal structure, and interaction networks. Casein exists in a supramolecular form as micelles [59]. Internally, colloidal calcium phosphate clusters and phosphorylated casein form a stable network through ionic interactions, placing certain sites like lysine in spatially constrained or shielded states. This reduces the probability of reducing sugars approaching and reacting with these sites. The direct comparison between the model system and skim milk powder indicates that the complex microstructure of skim milk powder reduces the magnitude of DPPH radical-scavenging activity [60].

## 5. Maillard Reaction Products and Antioxidant Potential

### 5.1. Melanoidins

The MRPs identified in dairy products with demonstrated antioxidant potential primarily include melanoidins, reduction ketones, and volatile heterocyclic compounds. Melanoidins are brown, nitrogen-containing, high-molecular-weight polymeric compounds [5,19]. The formation of melanoidins originates from highly reactive Maillard intermediates undergoing complex condensation, dehydration, and polymerization processes. Their generation is strongly influenced by the types of substrates and reaction conditions, although the exact formation mechanisms have not yet been fully elucidated. High-molecular-weight MRPs (melanoidins) formed in the final stage of the MR contribute to antioxidant activity through the ability to scavenge free radicals and bind metal ions [19,61,62,63,64]. The antioxidant activity of MRPs varies significantly with their molecular weight. High-molecular-weight MRPs exhibit particularly strong metal-chelating ability, a property facilitated by their richer composition of hydroxyl [9,65].

### 5.2. Reductones

The ε-amino groups of lysine residues in milk proteins react with lactose to form Amadori products. Under near neutral pH conditions, Amadori compounds preferentially undergo the 2,3-enolization reaction [1]. The 1,4-glycosidic bond structure in lactose affects subsequent reaction pathways, resulting in differences from glucose and other monosaccharides. During reaction, deoxyfuranone intermediates derived from disaccharides and monosaccharides undergo cyclization and enolization to form five-membered pyranone and six-membered furanone ring structures, respectively. These intermediates subsequently cleave to generate reductones and cyclic dicarbonyl compounds [66]. Reductones exert antioxidant effects by donating hydrogen atoms to terminate free radical chain reactions. They can also react with precursors of peroxides, thereby preventing peroxide formation and contributing to the overall antioxidant capacity [67]. Meanwhile, lactose may undergo the 4-deoxyosone pathway, resulting in the removal of the galactosyl moiety and formation of amino reductone structures bound to milk proteins [30]. In the study conducted by Tomoko Shimamura, the presence of amino reductones formed during the MR in milk was directly confirmed using a combination of 2,4-dinitrophenylhydrazine (DNP) derivatization and Cu^2+^ complexation techniques [68]. Although these compounds have been reported to exhibit antioxidant activity, they are difficult to extract completely from milk proteins [30,69]. Nevertheless, Shimamura successfully isolated a DNP-derivatized amino reductone oxidized by Cu^2+^ and elucidated its detailed molecular structure using NMR spectroscopy [68].

### 5.3. Volatile Heterocyclic Compounds

The MR not only generates antioxidant compounds such as melanoidins and reductones but also produces volatile heterocyclic MRPs that contribute to both flavor and antioxidant activity. GC–MS analysis has revealed that these volatile heterocyclic MRPs primarily consist of oxygen-, nitrogen-, and sulfur-containing heterocyclic compounds, including oxygen heterocycles such as furans, nitrogen heterocycles such as pyrazines, and sulfur heterocycles such as thiophenes and thiazoles [70]. The antioxidant activity of these compounds is attributed to the presence of aromatic and heterocyclic structures (e.g., pyrazine and furan rings), carbonyl groups (originating from Amadori products), phenolic groups, as well as amine and amide functionalities. These groups can donate electrons or chelate metal ions, thereby neutralizing free radicals and reactive oxygen species (ROS). However, their overall contribution to antioxidant capacity is generally lower than that of melanoidins [19].

The strength of antioxidant activity of volatile heterocyclic compounds is closely related to the electron density of the heterocyclic ring. Pyrrole, furan, and thiophene rings possess relatively high π-electron cloud densities on their five-membered ring carbons, facilitating electrophilic addition reactions and resulting in stronger antioxidant properties. The presence of heteroatoms like sulfur or nitrogen in thiazole and pyrazine rings reduces the π-electron density, hindering electrophilic addition reactions and leading to weaker antioxidant activity [71]. A study assessed the antioxidant activity of 12 heterocyclic intermediates derived from the MR. It found that several compounds, including pyranones and pyridones, showed antioxidant activity similar to ascorbic acid [72]. Furthermore, the specific type of volatile MRP formed can be selectively influenced by the choice of amino acid and sugar, as well as reaction temperature and duration. In the study by Cho et al., a comparison of volatile MRPs derived from reactions between different reducing sugars and amino acids (including furans, pyrazines, and sulfur-containing thiazoles) demonstrated that ketose sugars (such as fructose and tagatose) produced a greater abundance of furans and their derivatives compared with aldoses (such as glucose and galactose) [73].

## 6. Factors Influencing the Antioxidant Activity of Maillard Reaction Products

### 6.1. Substrate Type and Proportion

#### 6.1.1. Protein and Amino Acids

Dairy products contain a variety of proteins and amino acids, with proteins primarily composed of caseins and whey proteins [74]. Casein, a phosphoprotein with calcium-binding capacity, accounts for approximately 80% of total milk proteins and contributes significantly to whole milk’s antioxidant capacity [48,75]. The antioxidant activities of casein-derived MRPs have been extensively investigated [76,77]. MRPs generated from the yak casein–glucose system exhibit higher antioxidant activity than those from the Holstein casein–glucose system, a difference attributed to the compositional variations in the protein sources [9]. The DPPH radical-scavenging rates of buffalo milk after boiling (30.4 ± 0.94%) and pasteurization (31.5 ± 0.67%) were significantly higher than those of cow milk treated under the same conditions (boiling: 23.6 ± 0.58%; pasteurization: 23.8 ± 1.10%) [4]. These results indicate that buffalo milk exhibits stronger antioxidant activity than cow milk, highlighting the influence of the protein source on antioxidant performance. In addition, A. Zulueta reported a significant positive correlation between fat content and total antioxidant capacity, suggesting that differences in milk fat levels may also contribute to the superior antioxidant properties of buffalo milk [48].

The antioxidant potential of whey protein arises largely from the metal-chelating activities of serum albumin and lactoferrin, as well as the radical-scavenging properties of amino acids such as tyrosine and cysteine [6,78]. α-Lactalbumin (α-LA) constitutes about 20–25% of whey proteins and is characterized by a high abundance of lysine residues (lysine accounts for 9.8% of its total amino acids) [79]. The MR induces unfolding of the α-LA structure and exposure of hydrophobic groups, making it more reactive and thus more prone to form antioxidant MRPs compared with other milk proteins [13,80,81]. β-Lactoglobulin (β-LG), representing 40–50% of total whey proteins [81], is relatively heat-labile and easily denatured; blockage of its thiol groups may lead to a loss of antioxidant activity [3].

The ε-amino group of lysine residues in proteins exhibits particularly high reactivity of MR [5,26]. In addition, the imidazole and indole groups present in histidine and tryptophan, respectively, as well as the α-amino groups of terminal amino acids in proteins or peptides, can also participate in the reaction to a lesser extent [28,82]. The active participation of arginine, histidine, and lysine residues in the MR partially explains the superior ability of whey proteins to generate antioxidant MRPs [10].

Reaction reactivity is typically ranked in the following order: amines > amino acids > proteins [1]. This reactivity order explains why enzymatic hydrolysis of proteins are often employed before the MR. Hydrolysis releases peptides and free amino acids, exposing more reactive amino groups and enhancing the antioxidant potential of the MRPs. This effect has been confirmed in both whey protein hydrolysate and casein hydrolysate model systems [35,83,84].

#### 6.1.2. Reducing Sugars

The sugar composition of dairy products is primarily lactose, along with glucose, sucrose, fructose, maltose, and galactose [10]. The general order of reducing sugar reactivity follows the order: reducing pentoses > reducing hexoses; monosaccharides > oligosaccharides; aldohexoses > ketohexoses > disaccharides [1,34,85]. For instance, the antioxidant activity of casein–glucose MRPs is generally higher than that of casein–lactose MRPs [86]. This difference arises mainly from the smaller molecular size of monosaccharides, which allows better accessibility of reactive sites and facilitates collisions with amino groups of proteins. The same phenomenon was verified in the lactose/glucose-whey protein system, but the lactose system yielded higher compound concentrations. Prolonging the incubation time may further enhance MRP formation in the lactose system [5]. Two studies by Hao Zhang indicate that the FRAP value of α-LA is approximately 2 μmol ascorbic acid/g protein. Upon addition of sugar, the value increased twenty-five-fold for α-LA-polydextrose and six-fold for α-LA-2′-fucosyllactose [13,80]. Moreover, the α-LA-fructose system exhibits stronger reducing capacity than the α-LA-fructo-oligosaccharides system—demonstrating the superior reactivity of monosaccharides as glycosylation substrates compared to higher-polymerized oligosaccharides [79].

#### 6.1.3. The Effect of the Sugar-Protein Ratio

In casein–sugar systems, the relative ability of glucose and lactose to induce antioxidant activity depends on the concentration of each reactant and the casein-to-sugar ratio. The highest antioxidant activity was obtained when a mixture containing 10.0% casein and 2.5% glucose was heated. In comparison, a similar concentration of mixture (10.0% casein and 2.5% lactose) also yielded maximal protective index (PI) under identical conditions. PI = 1 indicates no antioxidant effect, PI > 1 an antioxidant effect, and PI < 1 a pro-oxidant effect [86]. In yak milk, the radical-scavenging activity of MRPs was maximized at a glucose-to-casein ratio of 1.5:1 (*w*/*w*), whereas the reducing power of Holstein MRPs was highest at different glucose-to-casein concentrations with the same ratio (Table 4) [9]. Moreover, response surface methodology indicated that a substrate concentration of 20%, a casein-to-glucose ratio of 1:2, a heating time of 132.7 min, a temperature of 100.2 °C, and an initial pH of 12.0 were the optimal conditions for enhancing MRP antioxidant activity [87]. The core reason why the “optimal casein-to-glucose ratio” varies across different studies lies in the differing research objectives and evaluation systems. The Maillard reaction is influenced by reaction conditions. When optimizing via response surface methodology, the optimal solution is the combined result of “ratio × pH × time × temperature.” However, Barry J. McGookin only discussed the optimal “casein-to-glucose ratio” under different ratio conditions. Additionally, each antioxidant evaluation method focuses on different aspects of the detection mechanism, leading to non-overlapping “strongest antioxidant activity” results for the same sample across different indicators.

### 6.2. Reaction Conditions

#### 6.2.1. Process Technology

To ensure the microbial safety and extend the shelf life of dairy products, thermal processing remains a conventional and widely applied technology. Non-thermal processing technologies (including high hydrostatic pressure (HHP), pulsed electric fields (PEF), ultrasound, and UV-C irradiation) also can effectively inactivate microorganisms while minimizing Maillard reactions, thereby better preserving freshness, nutritional value, and the original color and flavor [88]. Heat treatment primarily relies on temperature-driven kinetics to promote the carbonyl-amino condensation reaction. Elevated temperatures increase molecular kinetic energy [89], driving rearrangement or polymerization of substances in the final stages of the MR to generate antioxidant-active MRPs. In contrast, most non-thermal techniques modify antioxidant benefits by altering protein conformation to expose reactive groups or through disulfide bond rearrangement [90]. Ultrasonication minimally affects the secondary and tertiary structures of most proteins and does not break covalent bonds. However, ultrasonic treatment of β-LG in bovine milk significantly enhances its antioxidant activity [91]. Ultrasonic treatment causes β-LG to adopt a looser conformation, reducing α-helix content and altering secondary structure. It increases the protein’s β-sheet and ring-like structures, expanding surface area and exposing more radical-reactive sites [92,93,94]. The presence and positioning of amino acids within β-LG determine its antioxidant activity [95]. Compared to the control group’s DPPH value (32.27 ± 0.70%) and ABTS value (8.51 ± 0.49%), UV-C treatment in 30 min significantly enhanced antioxidant activity of β-LG (*p* < 0.05). The DPPH value nearly doubled (62.59 ± 0.83%), and the ABTS value increased tenfold (80.30 ± 0.12%) [95]. Furthermore, current research has not extensively addressed the comparison of antioxidant capacity between heat-induced and non-heat-induced dairy products. Future studies should evaluate the advantages and disadvantages of thermal and non-thermal processing technologies in terms of antioxidant efficacy, retention of heat-sensitive nutrients, and generation of harmful substances.

#### 6.2.2. Reaction Temperature

The impact of temperature on the antioxidant activity of MRPs has been widely reported. Numerous studies indicate that appropriately increasing temperature typically enhances the reactivity between sugars and amino acids, thereby accelerating the MR and boosting the antioxidant activity of MRPs [96]. Diego A. Cortés Yáñez observed in a whey protein–lactose model system that DPPH radical scavenging activity increased linearly with temperature from 37 °C to 60 °C, indicating that higher temperatures promote the formation of antioxidant compounds [5]. During this process, milk proteins also undergo denaturation, causing globular structures to unfold and exposing internal cysteine residues and sulfhydryl groups. This process reduces the redox potential of the dairy system [34]. Another study comparing untreated with moderate/strong thermal processing found that treatment at 90 °C for 30 min resulted in a significantly higher ferric-reducing power than other conditions (*p* < 0.05), with values increasing progressively as the intensity of heat treatment increased [97]. However, temperatures exceeding 200 °C may cause the degradation and denaturation of MRPs, leading to a reduction in their antioxidant properties [19].

#### 6.2.3. Reaction Time

Reaction time directly influences the extent of the MR as well as the concentration of antioxidant-active compounds. Insufficient reaction time may result in the inadequate accumulation of MRPs, whereas excessively prolonged reactions can lead to the degradation or polymerization of products, potentially reducing antioxidant activity or generating undesirable by-products. In a xylose–casein hydrolysate model system, Jiang et al. observed that extending the heating time from 0 to 8 h gradually enhanced both DPPH radical scavenging activity and ferrous ion reducing power, indicating that prolonged reaction time favors the formation and transformation of antioxidant compounds [35]. Liu et al. reported in a dry-heated whey protein–glucose system that increasing the reaction duration from 0 to 7 days significantly improved the reducing power and ABTS radical scavenging activity of the MRPs (*p* < 0.05) [98]. Additionally, antioxidant activity generally stabilizes after about 48 h of reaction at 60 °C, indicating the potential for reaction equilibrium or product degradation [13]. Therefore, precise control of reaction time is crucial for maximizing the antioxidant potential of MRPs.

#### 6.2.4. pH

PH is a key factor determining the MR pathway. It influences the ionization state of reactants and reaction rate, thereby dictating the reaction pathway and the types of MRPs formed. Under alkaline conditions (pH 8–9), the ε-amino groups of lysine in proteins are deprotonated, enhancing their nucleophilicity and significantly accelerating the early stage of the MR [55]. The reaction rate of MR typically rises as pH (3–9) and temperature increase, and antioxidant production tends to be more efficient in alkaline environments [1,55,99]. And the high-molecular-weight MRP generated by the casein-glucose model system under alkaline conditions exhibits enhanced metal chelation capacity [76]. Under acidic to neutral conditions (pH 3–7), the reaction pathway tends to 1,2-enolization, which preferentially produces furan derivatives such as HMF. These compounds typically exhibit weaker antioxidant activity [10,26]. In lactose–lysine model systems, different pH conditions can lead to the formation of intermediates such as 1-deoxyglycose, 3-deoxyglycose, or 4-deoxyglycose, which affect the antioxidant activity of MRPs [29]. Moreover, the accumulation of organic acids such as formic and acetic acids during the MR can lower the system pH, further affecting reaction progression and product distribution [35,44]. Notably, the antioxidant capacity of unheated casein–sugar systems is largely unaffected by the initial pH. Upon heating, casein–glucose or casein–lactose mixtures at an initial pH of 6.8 show increased antioxidant activity, whereas at higher initial pH values of 7.8 or 8.8, heating does not result in significant changes in the PI [86]. Under a fixed glucose or lactose concentration, the pH change during heating is independent of casein concentration and depends primarily on the initial molar concentration of sugar in the casein–sugar mixture [86]. Similar findings were reported by Haixia Wang, confirming the pH-dependence of MRPs formation and antioxidant properties [9].

#### 6.2.5. Water Activity

Water activity (aw) varies considerably among different dairy products. Fluid products such as milk and dairy beverages exhibit high water content (aw > 0.95), whereas low-moisture products such as milk powder and ghee have much lower water activity (aw < 0.2) [26]. Multiple dehydration processes and many MR pathways produce additional water molecules. Water activity directly influences the kinetics of the MR by regulating the mobility and solubility of reactants as well as the viscosity of the reaction medium. The optimal water activity range for generating MRPs is 0.6–0.8. Within this range, reactants have enough mobility to interact with each other. Simultaneously, the water content remains sufficiently low to prevent excessive dilution of intermediate products [5]. Under such conditions, the reaction rate generally increases with water content [1]. In investigating the effect of temperature on a whey protein–lactose system, Diego A. Cortés Yáñez adjusted the water activity to 0.52 to ensure efficient reaction progression [5]. During the production of spray-dried milk powders, controlling water activity is an effective strategy to regulate the extent of the MR. Research indicates that in spray-drying processes with inlet temperatures of 190–250 °C, appropriate water activity control stabilizes the secondary structure of whey proteins while simultaneously promoting the formation of MRPs, thereby enhancing the antioxidant capacity of the final product [100].

**Table 4:** MR conditions and main findings are summarized.

Factor	Sample	Reaction Conditions	Main Findings	References
Protein	whey protein isolate (WPI) and whey protein hydrolysates (WPHs), Galactose.	95 °C for 0 h, 1 h, 2 h, 3 h, and 4 h.	WPI-Gal and WPH-Gal conjugates showed ABTS•^+^ radical scavenging activity from5.50% to 46.29% and 17.41% to 69.81%. Whey protein hydrolysates exhibited higher antioxidant activity.	[83]
Protein	Yak casein-glucose, Holstein casein-glucose.	100 °C, 3 h.	The antioxidant activity of the Yak casein-glucose system exhibited higher reducing power and DPPH radical scavenging activity than the Holstein casein-glucose system.	[9]
Protein-to-sugar ratio	Yak/Holstein glucose was mixed with the casein dispersion according to the following ratios: glucose/casein (*w*/*w*) at 0:1, 0.5:1, 1:1, 1.5:1, 2:1, 2.5:1.		The antioxidant activity of the Yak casein-glucose system increased with rising glucose content and, under identical conditions.	[9]
Reducing sugars	alpha-lactalbumin (α-LA), fructose and fructo-oligosacchrides (FOS)	60 °C for 12 and 24 h (FOS-based MRPs) or 12 and 36 h (fructose-based MRPs).	There was no significant difference in FRAP values between α-LA-fructose 12 h and α-LA-FOS 12 h (*p* > 0.05), but the FRAP value of α-LA-fructose 36 h was significantly greater than that of α-LA-FOS 24 h (*p* < 0.05).	[79]
Reaction temperature	Camel milk	The treatments ranged from no thermal treatment to moderate (63 °C for 30 min and 72 °C for 15 s) and high (85 °C for 15 s and 30 min, and 90 °C for 15 s and 30 min) thermal treatments.	Samples from milk heated at 90 °C exhibited the highest DPPH scavenging activity and reducing power (*p* < 0.05).	[97]
Reaction time	Casein, xylose	Thermal treatment of 0, 1, 2, 3, 4, 5, 6, 7 and 8 h a t 100 °C in an oil bath.	Both the DPPH radical scavenging capacity and ferrous reducing activity of MRPs showed a progressive increase throughout the 8 h reaction.	[35]
pH	Casein-lactose mixtures, casein-glucose mixtures, casein solutions without added sugar.	The pH was adjusted to 6.8, 7.8, and 8.8, respectively. 2 h at 120 °C in a glycerol bath.	The antioxidant capacity of the unheated casein-sugar system was not affected by the initial pH. When the pH of the casein solution was 6.8, heating led to an increase in the antioxidant capacity of the casein-glucose/lactose system, whereas no substantial change in the PI was observed during heating at casein solution pH values of 7.8 or 8.8 (*p* > 0.05).	[86]

ABTS•^+^ refers to the ABTS radical cation.

## 7. Application of the Maillard Reaction to Enhance Antioxidant Activity in Dairy Products

### 7.1. Maintaining the Quality of Dairy Products and Extending the Shelf Life of Dairy Products

**Figure 3:**
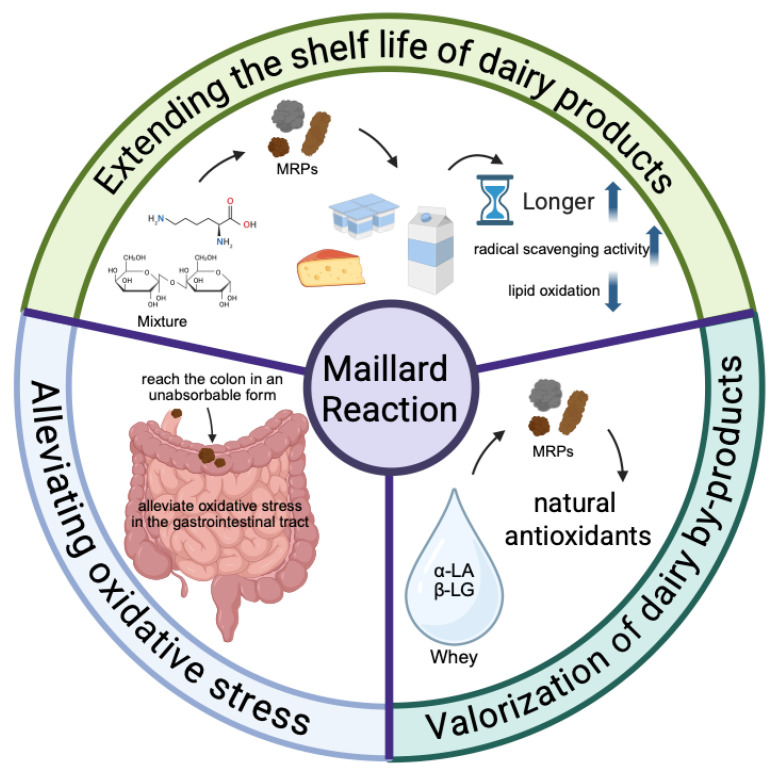
Applications of the antioxidant effects of MRPs in dairy products.

Protein/amino acid-sugar mixtures demonstrate excellent antioxidant properties in model systems. Upon storage at 5 °C for six days, the early-stage MRPs produced via the glucose-lysine system retained its antioxidant activity. The degradation level of methyl linolenate (13.3 ± 1.26%) was significantly lower than that of the control group (68.3 ± 5.26%) [101]. Glycosylation-modified β-LG exhibits enhanced radical scavenging activity and microbial growth inhibition [102]. Researchers have also attempted to apply MRPs to dairy products to investigate their impact on the products. It was found that the addition of pre-formed MRPs (casein peptide-glucose conjugates) into milk powder formulations effectively inhibited lipid oxidation, extended shelf life, reduced bitterness, and improved sensory properties (Figure 3) [103,104]. Furthermore, glycosylation of WPI with D-allose and D-psicose enhanced its emulsifying capacity, foam stability, and antioxidant activity. When applied to ice cream production, this modified protein exhibits an ABTS radical scavenging activity of approximately 80% [105]. In a related study on dairy-based beverages, the addition of heated milk protein–sugar mixtures as antioxidants effectively prevented the oxidation of polyunsaturated fatty acids in flaxseed oil-enriched drinks during sterilization [34]. Furthermore, the incorporation of glycine–glucose MRPs in margarine has been shown to improve its oxidative stability [106]. The aforementioned studies predominantly employed protein/amino acid-sugar mixtures as research subjects to investigate their beneficial effects on dairy products, yet failed to specify the particular MRPs responsible for their antioxidant activity.

However, potential drawbacks must be considered when applying MRPs to enhance dairy products’ antioxidant activity. Potential adverse effects include loss of essential nutrients like lysine, uncontrolled browning, reduced solubility, formation of off-flavor compounds, and generation of harmful substances [107]. During the final stages of the MR in dairy products, not only melanoidins are produced, but also advanced AGEs such as CML and CEL. Long-term dietary intake of AGEs may be associated with human aging and the development of chronic diseases such as diabetes, chronic kidney disease, osteoporosis, and Alzheimer’s disease [7,108,109]. In terms of processing intensity, mild heat treatments (such as pasteurization) typically cause only minor changes in dairy products’ antioxidant indicators, while the increase in AGEs remains relatively limited [4,110,111,112]. In contrast, intense heat treatments (such as UHT or sterilization) may cause a significant increase in antioxidant activity. As shown in Table 5, raw milk subjected to sterilization at 121 °C for 10 min exhibited significantly higher antioxidant index values than pasteurized milk treated at 63 °C for 30 min, with both exceeding the levels found in raw milk. Furthermore, Pamela Manzi found that the ultra-high-temperature (UHT) milk’s FRAP and DPPH values were significantly higher than those of the high-quality pasteurized (HQ) milk group (*p* < 0.05) [113]. However, this intense heat treatment was accompanied by a concurrent increase in AGEs, such as CML and CEL. The study revealed that after treatment at 121 °C for 25 min, the CML content in raw milk increased to 1.6 times the original level, while CEL content rose from 0.35 ± 0.02 mg/kg milk to 0.84 ± 0.02 mg/kg milk (Table 6) [112]. Infant formula milk powder produced via spray drying contains free and bound CML, CEL and pyroxalate, with free AGEs levels ranging from 0.19–2.79 mg/kg and bound AGEs levels from 35.20–75.02 mg/kg [107,114]. There is currently no internationally agreed standard for the safe intake range of AGEs. Therefore, when evaluating the application value of MRPs for enhancing the antioxidant activity of dairy products, their potential risks should also be considered.

The adverse effects of the MR include nutrient loss and the formation of potentially harmful compounds during thermal processing [96]. Non-thermal technologies offer alternatives to address these challenges. High-intensity ultrasound can induce the MR in whey protein–sugar systems, producing MRPs with antioxidant activity similar to that of thermal treatments when applied under specific conditions [115]. Ultrasonic treatment facilitates protein glycosylation under mild conditions and short reaction times, improving the efficiency of the early stage while minimizing excessive browning in the final stages [85]. Zhanwu Sheng et al. explicitly highlighted the potential of non-thermal techniques (high-pressure processing, pulsed electric fields, ultrasound, and cold plasma) to reduce AGEs. Concurrently, the formation of AGEs can be effectively controlled through a series of regulated treatments that modulate temperature, duration, and other critical parameters [116].

It is important to note that the shelf life of liquid dairy products remains largely controlled by microbial growth rather than solely dependent on the oxidation process itself. The “antioxidant gain and quality stability” resulting from high-temperature processing should be understood as one of the additional benefits of MR, but it also carries the risk of generating harmful substances. The “benefit-risk” balance between these two aspects requires careful consideration within the legally established safe intake range for AGEs. Future efforts should focus on refining relevant standards to ensure the safe application of naturally occurring antioxidants in dairy products and maintain food quality and safety. Additionally, to minimize AGE burden while ensuring safety, alternative processing methods or combined techniques may be considered. This approach aims to develop shelf-life extension strategies that simultaneously reduce AGE formation.

### 7.2. Alleviating Oxidative Stress in the Human Gastrointestinal Tract

Milk and dairy products (such as yogurt and cheese) constitute approximately 25–30% of the daily human diet and represent a significant source of natural antioxidant components [3]. As natural antioxidants, MRPs not only effectively inhibit protein and lipid oxidation but may also help reduce the risk of lifestyle-related diseases—such as cardiovascular diseases, cancer, diabetes, and obesity—when consumed through dairy products [3,54]. In fermented items such as yogurt and cheese, MRPs have been shown to offer multiple potential health benefits [3]. Pre-fermentation heat treatment of camel milk not only shortens fermentation time and increases the viscosity of the final product but also positively influences free radical scavenging activity and reducing power [97]. Research on fermented skim milk also indicates that MRPs offer potential health benefits for human consumption. Certain radical-scavenging compounds reached the colon in an unabsorbable form, contributing to protection against gastrointestinal oxidative stress [117]. The combination of heat treatment and fermentation serves as an effective strategy to enhance the antioxidant capacity of dairy protein systems, providing both a theoretical basis and practical potential for developing functional fermented dairy products with specific health benefits. Following the MR that links α-lactalbumin with polydextrose (PD), both antioxidant capacity and protein stability are significantly improved. This compound shows potential for developing into a carrier for proteins or prebiotics [13]. Yusuf Jubran et al. reported that, under simulated adult and infant digestive conditions, fructose or oligofructose induced α-LA glycosylation and delayed protein degradation, suggesting that this substance may help counteract oxidative reactions within the intestinal lumen [79].

### 7.3. Valorization of Dairy By-Products

Growing consumer demand for cheese and related dairy products has led to a substantial increase in whey production [118]. As a by-product of dairy processing, approximately 30–35% of whey is discarded. However, whey protein has been scientifically confirmed to possess antioxidant activity, making it a high-quality raw material for preparing natural antioxidants [54,119]. One study specifically evaluated the MR potential of whey from different sources—bovine cheese whey, caprine cheese whey, and bovine yogurt whey. After heating at 140 °C for 90 min, all types of whey generated known antioxidative MRPs, including 2-pyrrolecarboxaldehyde and maltol isomer, among 28 key compounds. Bovine cheese whey contains the most diverse array of antioxidants [10]. Another reported that MRPs made from whey protein concentrate (WPC), sodium caseinate (SC), and lactose synergistically enhanced antioxidant effects and improve functional properties during fermentation, showing potential for reducing cardiovascular disease risk [49]. The Maillard reaction transforms whey into functional products with antioxidant activity, offering novel approaches for the resourceful reuse of by-products.

The application of MRPs from whey in industrial production faces numerous constraints, such as reproducibility, cost control, and product acceptance. Due to the sensitivity of the MR to reaction conditions, scaling up from laboratory conditions to industrial applications poses challenges in precisely controlling reaction temperatures. Heating processes may cause uneven sample heating, with localized overheating creating hot spots that lead to undesirable outcomes [85]. Although whey raw materials themselves are relatively inexpensive, overall production costs primarily stem from energy consumption, precise reaction condition control, and the separation and extraction of antioxidants. These operations aim to reduce off-flavors like “burnt” and mitigate the impact of natural browning on the appearance and consumer acceptance of dairy products. However, operations like decolorization increase process complexity, partially offsetting the cost advantage of low-cost raw materials. Furthermore, most current research involves adding mixtures of proteins/amino acids and carbohydrates to dairy products. As structurally complex crude products, these mixtures currently lack efficient methods for separating effective antioxidant components. Utilizing MRPs as natural antioxidants in food also necessitates consideration of food safety concerns and consumer acceptability.

## 8. Conclusions and Future Work

Overall, evidence from the included studies indicates that MRPs derived from dairy products exhibit measurable antioxidant activity, manifested in DPPH radical scavenging, reduction of ABTS•^+^ radical cation absorbance, and enhanced iron ion reduction capacity in the FRAP system. Due to variations in dairy matrices, reaction conditions, and antioxidant assay methods, the magnitude and comparability of antioxidant effects differed across studies. The antioxidant activity of MRP not only extends the shelf life of dairy products and enhances their sensory quality but also alleviates oxidative stress in the human gut. Furthermore, producing natural antioxidants from the industrial by-products whey demonstrates promising application potential. However, current research has yet to fully elucidate the precise composition, structural characteristics, and antioxidant mechanisms of MRPs, which to some extent limits the production and application of natural antioxidants. Therefore, future research should focus on clarifying the structure–activity relationships of MRPs, elucidating their antioxidant mechanisms, and developing efficient and precisely controllable processing techniques. While enhancing the antioxidant performance of MRPs, attention must also be given to suppressing the formation of harmful by-products generated during the MR, so as to promote their safe and effective application in the dairy processing industry.

## Figures and Tables

**Table 1:** A critical comparative table.

Review	Year and Journal	Keywords	Main Focus	Differential Contribution of Our Review
Magdalena Stobiecka et al. [3]	2022, animals	Milk; dairy products; bioactive compounds; bioactive peptides; total antioxidant capacity	This review examined the antioxidant potential of natural antioxidants in raw milk and dairy products. It summarized potential pathways for regulating and enhancing antioxidant levels throughout the milk production chain.	This review did not focus on the MR. In contrast, our review focused on the antioxidant activity of MRPs within dairy systems under MR conditions.
Mingyu Li et al. [18]	2022, Food Research International	Maillard reaction; Dairy products; Furosine; Furfurals; Advanced glycation end products	This review covers the harmful MRPs formation profiles and content levels in dairy products, detection methods, toxicity risks, and feasible control and mitigation strategies.	Our review also briefly discussed the formation of harmful products from the MR under specific conditions, but primarily focused on the antioxidant activity of MRPs.
Sara Bolchini et al. [19]	2025, European Food Research and Technology	Maillard reaction products; Antioxidant activity; Food preservation; Sustainable utilization; Food waste valorization	This review discussed the antioxidant properties of MRPs in food, the principles of detection methods, and their potential as natural food preservatives.	Unlike the foods examined in this review, our review focuses on dairy products.
Leina El Hosry et al. [20]	2025, foods	S-Maillard reaction; non-enzymatic browning; vitamin C degradation; acrylamide; melanoidins; advanced glycation end-products; nutritional effect; food processing	This review provided a systematic and comprehensive summary of the mechanisms, influencing parameters, benefits, and drawbacks of the Maillard reaction in food, as well as its applications in the food industry.	Our review summarized the pathways of MR in dairy products and the antioxidant potential of MRPs derived from different components. Additionally, it discussed the industrial applications of dairy-derived MRPs.

**Table 2:** Search terms and strategy by database.

Concept	Principle
Dairy products	“milk”, “buffalo milk”, “Holstein cow milk”, “human milk”, “yogurt”, “milk powder”, “cheese”, “whey protein”, “casein”, “β-LG”, “α-LA”, “lysine”, “lactose”, “glucose”, “galactose”, “fructose”
Maillard reaction	“Maillard reaction”, “non-enzymatic browning reaction”
Antioxidant activity	“antioxidant activity”, “antioxidant”

**Table 3:** Determination of antioxidant activity in dairy products [3].

Method	Principle	Observation
FRAP	The antioxidant capacity was monitored by measuring the reduction of the iron (III) complex with 2,4,6-tris(2-pyridyl)-1,3,5-triazine ([Fe^3+^-(TPTZ)_2_]^3+^) to the intense blue iron (II) complex ([Fe^2+^-(TPTZ)_2_]^2+^)	Spectrophotometric analysis at 593 nm
DPPH	Antioxidant compounds reduce the purple-colored, 2,2-diphenyl-1-picrylhydrazyl (DPPH) radical to yellow 2,2-diphenyl-1-picrylhydrazine	Visual assessment or spectrophotometric analysis at 517 nm
ABTS	Antioxidants lead to the reduction of the cation radical ABTS+—2,2-azinobis-(3-ethylbenzothiazoline-6-sulfonate, causing discoloration of the blue-green solution	Visual assessment or spectrophotometric analysis at 734 nm

**Table 5:** Evaluation of antioxidant activity in heat-treated cow’s milk.

Sample	Heating Conditions	DPPH	ABTS	Reference
Raw cow milk	-	19.16 ± 0.08 ^Ba^	48.66 ± 3.27 ^Aa^	[111]
Pasteurized cow milk	63 °C 30 min	21.81 ± 0.15 ^Ba^	47.99 ± 2.81 ^Aa^	
Sterilized cow milk	121 °C 10 min	43.11 ± 1.56 ^Aa^	68.46 ± 3.22 ^Ab^	
Raw sheep milk	-	24.62 ± 1.21 ^Ca^	59.57 ± 3.47 ^Ba^	
Pasteurized sheep milk	63 °C 30 min	25.82 ± 1.19 ^Ca^	66.44 ± 3.14 ^Bb^	
Sterilized sheep milk	121 °C 10 min	80.41 ± 2.54 ^Cb^	74.35 ± 0.96 ^Ac^	
Raw goat milk	-	13.34 ± 0.27 ^Aa^	61.31 ± 3.22 ^Ba^	
Pasteurized goat milk	63 °C 30 min	16.17 ± 0.30 ^Ab^	65.60 ± 3.94 ^Bab^	
Sterilized goat milk	121 °C 10 min	73.12 ± 1.04 ^Bc^	73.58 ± 4.20 ^Ab^	
Raw cow milk	-	24.3 ± 0.49 ^d^	-	[4]
Pasteurized cow milk	65 °C 30 min	23.8 ± 1.10 ^c^	-	
Boil cow milk	boiling for 1 min	23.6 ± 0.58 ^c^	-	
Raw buffalo milk	-	31.8 ± 1.77 ^a^	-	
Pasteurized buffalo milk	65 °C 30 min	31.5 ± 0.67 ^a^	-	
Boil buffalo milk	boiling for 1 min	30.4 ± 0.94 ^a^	-	

^ABC^ Different uppercase letters show statistical differences of different ruminants in the same treated (*p* < 0.05), and ^abcd^ Different lowercase letters show statistical differences of each treated milk (*p* < 0.05).

**Table 6:** The concentration of AGEs in raw milk and with pasteurization and sterilization methods [112].

Sample	Heating Conditions	CML	CEL
Raw milk	-	2.36 ± 0.27 ^c^ mg/kg milk	0.35 ± 0.02 ^B^ mg/kg milk
Low-temperature long-time (LTLT)	65 °C 30 min	2.82 ± 0.27 ^b^ mg/kg milk	-
High-temperature short-time (HTST)	82 °C 15 s	2.70 ± 0.09 ^bc^ mg/kg milk	-
Ultra-high temperature (UHT)	137 °C 4 s	1.5-fold than that in raw milk (nearly 3.54 mg/kg milk)	-
In-bottle sterilization (BS)	121 °C 25 min	3.88 ± 0.10 ^a^ mg/kg milk	0.84 ± 0.02 ^A^ mg/kg milk

^AB^ Different uppercase letters show statistical differences of CEL (*p* < 0.05), and ^abc^ Different lowercase letters show statistical differences of CML (*p* < 0.05).

## Data Availability

No new data were created or analyzed in this study. Data sharing is not applicable to this article.

## Supplementary Materials

The following supporting information can be downloaded at: https://www.mdpi.com/article/10.3390/foods15020351/s1, Table S1. Quantification of articles based on basic and combined descriptors, searched on the PubMed. Table S2. Quantification of articles based on basic and combined descriptors, searched on the Web of Science.

## Abbreviations

The following abbreviations are used in this manuscript:

ABTS	2,2′-azino-bis (3-ethylbenzthiazoline-6-sulfonic acid
AGEs	Advanced glycation end-products
ARPs	Amadori rearrangement products
CEL	N-ε-(carboxyethyl)lysine
CML	N-ε-(carboxymethyl)lysine
DPPH	Diphenyl picryl hydrazhyl
FRAP	Ferric reducing antioxidant power
HMF	5-hydroxymethylfurfural
MRPs	Maillard reaction productsH
MR	Maillard reaction
PI	Protective index
UV	Ultraviolet
WPC	Whey protein concentrate
WPI	Whey protein isolate
α-CN	α-casein
α-LA	α-lactalbumin
β-LG	β-lactoglobulin
