# Supplementary material for: Antioxidant Activity of Maillard Reaction Products in Dairy Products: Formation, Influencing Factors, and Applications
Source: Foods. 2026 Jan 18;15(2):351. doi: 10.3390/foods15020351 (PMC12841591; doi:10.3390/foods15020351)
Supplement: Supplementary file 1 [file foods-15-00351-s001.zip › foods-4020029-supplementary.pdf]

Table S1. Quantification of articles based on basic and combined descriptors, searched on the PubMed.

Database	Descriptors and Boolean Operators	Number of Studies
**PubMed**	(("milk" OR "buffalo milk" OR "Holstein cow milk" OR "human milk" OR "yogurt" OR "milk powder" OR "cheese") OR (("whey protein" OR "casein" OR "β-LG" OR "α-LA" OR "lysine") AND ("lactose" OR "glucose" OR "galactose" OR "fructose")))	184,518
	((("milk" OR "buffalo milk" OR "Holstein cow milk" OR "human milk" OR "yogurt" OR "milk powder" OR "cheese") OR (("whey protein" OR "casein" OR "β-LG" OR "α-LA" OR "lysine") AND ("lactose" OR "glucose" OR "galactose" OR "fructose")))) AND ("Maillard reaction" OR "non-enzymatic browning reaction")	604
	((("milk" OR "buffalo milk" OR "Holstein cow milk" OR "human milk" OR "yogurt" OR "milk powder" OR "cheese") OR (("whey protein" OR "casein" OR "β-LG" OR "α-LA" OR "lysine") AND ("lactose" OR "glucose" OR "galactose" OR "fructose")))) AND ("Maillard reaction" OR "non-enzymatic browning reaction") AND ("antioxidant activity" OR "antioxidant")	74

Table S2. Quantification of articles based on basic and combined descriptors, searched on the Web of Science.

Database	Descriptors and Boolean Operators	Number of Studies
Web of Science	(("milk" OR "buffalo milk" OR "Holstein cow milk" OR "human milk" OR "yogurt" OR "milk powder" OR "cheese") OR (("whey protein" OR "casein" OR "β-LG" OR "α-LA" OR "lysine") AND ("lactose" OR "glucose" OR "galactose" OR "fructose")))	320192
	((("milk" OR "buffalo milk" OR "Holstein cow milk" OR "human milk" OR "yogurt" OR "milk powder" OR "cheese") OR (("whey protein" OR "casein" OR "β-LG" OR "α-LA" OR "lysine") AND ("lactose" OR "glucose" OR "galactose" OR "fructose")))) AND ("Maillard reaction" OR "non-enzymatic browning reaction")	1128
	((("milk" OR "buffalo milk" OR "Holstein cow milk" OR "human milk" OR "yogurt" OR "milk powder" OR "cheese") OR (("whey protein" OR "casein" OR "β-LG" OR "α-LA" OR "lysine") AND ("lactose" OR "glucose" OR "galactose" OR "fructose")))) AND ("Maillard reaction" OR "non-enzymatic browning reaction") AND ("antioxidant activity" OR "antioxidant")	146
